# Technological variability in the Late Palaeolithic lithic industries of the Egyptian Nile Valley: The case of the Silsilian and Afian industries

**DOI:** 10.1371/journal.pone.0188824

**Published:** 2017-12-27

**Authors:** Alice Leplongeon

**Affiliations:** 1 McDonald Institute for Archaeological Research, University of Cambridge, Cambridge, United Kingdom; 2 UMR CNRS 7194 HNHP, département Homme et Environnement, Muséum national d’Histoire naturelle–Université de Perpignan Via Domitia–Sorbonne Universités, Paris, France; 3 Institute of Archaeology, Hebrew University of Jerusalem, Jerusalem, Israel; University College Dublin, IRELAND

## Abstract

During the Nubia Salvage Campaign and the subsequent expeditions from the 1960’s to the 1980’s, numerous sites attributed to the Late Palaeolithic (~25–15 ka) were found in the Nile Valley, particularly in Nubia and Upper Egypt. This region is one of the few to have allowed human occupations during the dry Marine Isotope Stage 2 and is therefore key to understanding how human populations adapted to environmental changes at this time. This paper focuses on two sites located in Upper Egypt, excavated by the Combined Prehistoric Expedition: E71K18, attributed to the Afian industry and E71K20, attributed to the Silsilian industry. It aims to review the geomorphological and chronological evidence of the sites, present a technological analysis of the lithic assemblages in order to provide data that can be used in detailed comparative studies, which will allow discussion of technological variability in the Late Palaeolithic of the Nile Valley and its place within the regional context. The lithic analysis relies on the *chaîne opératoire* concept combined with an attribute analysis to allow quantification. This study (1) casts doubts on the chronology of E71K18 and related Afian industry, which could be older or younger than previously suggested, highlights (2) distinct technological characteristics for the Afian and the Silsilian, as well as (3) similar technological characteristics which allow to group them under a same broad techno-cultural complex, distinct from those north or south of the area.

## Introduction

The Nile Valley geographically links eastern Africa to North Africa and the Levant, and is therefore key in discussions of modern human dispersals out-of and back-into-Africa during the Upper Pleistocene [1–6]. However, the number, routes and timing of these dispersals are highly controversial [7]. Archaeological evidence supporting the ‘northern’ route out of Africa through the Nile Valley is sparse and debated ([8], but see [9,10]) and human remains from this period, all attributed to modern human remains, remain few [11–14]. Most of the evidence for Pleistocene dispersals thus comes from genetic results. Comparisons between the archaeological record of the Nile Valley and adjacent regions are at the heart of testing dispersal hypotheses and their archaeological visibility.

The Late Pleistocene (~75-15ka) corresponds to a period of major climatic changes, including a global decrease in precipitation. In northern Africa, this period is characterised by an oscillation between semi-arid and extremely arid conditions, the latter of which prevail particularly during the 'Last Glacial Maximum' (LGM, ~23-18ka). The Sahara expands, with only one wet phase identified (~50-45ka [15]), until the abrupt onset of the African Humid Period (~15ka [16]). The shift to more arid conditions is also associated with the lowering of sea level and the desiccation of some major eastern African lakes during the LGM (e.g. [17–19]). This has important consequences for the behaviour of the River Nile, its role as an ecological refugium, and on human populations living in its vicinity.

In a recent study, Vermeersch and Van Neer [20] argue that the number of radiocarbon dated sites, used as a proxy for population density, show two distinct periods of human occupations in the Upper Egyptian Nile Valley within MIS 2: one from 23 to 20 ka cal BP and another from 16 to 14 ka cal BP. This increase in the number of dated sites / population density also seems to correspond to an increase in the diversity of Late Palaeolithic cultural entities (e.g. [21,22]).

Most of these Late Palaeolithic industries were defined following the seminal work of the Combined Prehistoric Expedition in Nubia, Egypt and Sudan as part of the Nubia Salvage Campaign during the construction of the Aswan dam [21,23,24], as well as the work of a joint Yale University and Canadian team working in the Kom Ombo Plain [25–27]. Between the 1970’s and the 1990’s, the Combined Prehistoric Expedition worked mainly in Upper Egypt, while another major team, the Belgian Middle Egypt Prehistoric Project led by Prof. Vermeersch (Leuven University), worked in Middle Egypt [22,28]. In Egyptian Nubia and Upper Egypt, the principal cultural entities defined for the Late Palaeolithic are the Fakhurian, the Kubbaniyan, the Silsilian, the Idfuan-Shuwikhatian, the Afian and the Isnan [21,24].

One of the key questions of the Nile Valley archaeological record during the Late Pleistocene is how it relates to the archaeological record of adjacent areas (either south in the Upper Nile Valley in eastern Africa, or north in the Levant, or in northwestern Africa). Connections between the archaeological records of the Nile Valley and the Levant have been suggested [29,30], but no systematic comparative analyses of the lithic assemblages have been attempted. In addition, the lithic industries of the Late Palaeolithic of the Nile Valley were mostly defined based on typological criteria which prevents any thorough cross-regional comparisons between the different industries and assemblages.

This paper aims to contribute to facilitating these comparisons by conducting a comparative technological analysis of the lithic assemblages attributed to two of these Late Palaeolithic industries: E71K18C (attributed to the Afian industry) and E71K20 (attributed to the Silsilian industry) in order to situate them in the broader context of Late Pleistocene regional technological variability. This will help towards building a body of data that can be used in further comparative analysis with adjacent areas.

## Presentation of the sites and of the industries

### The Afian industry and E71K18C

**Fig 1:**
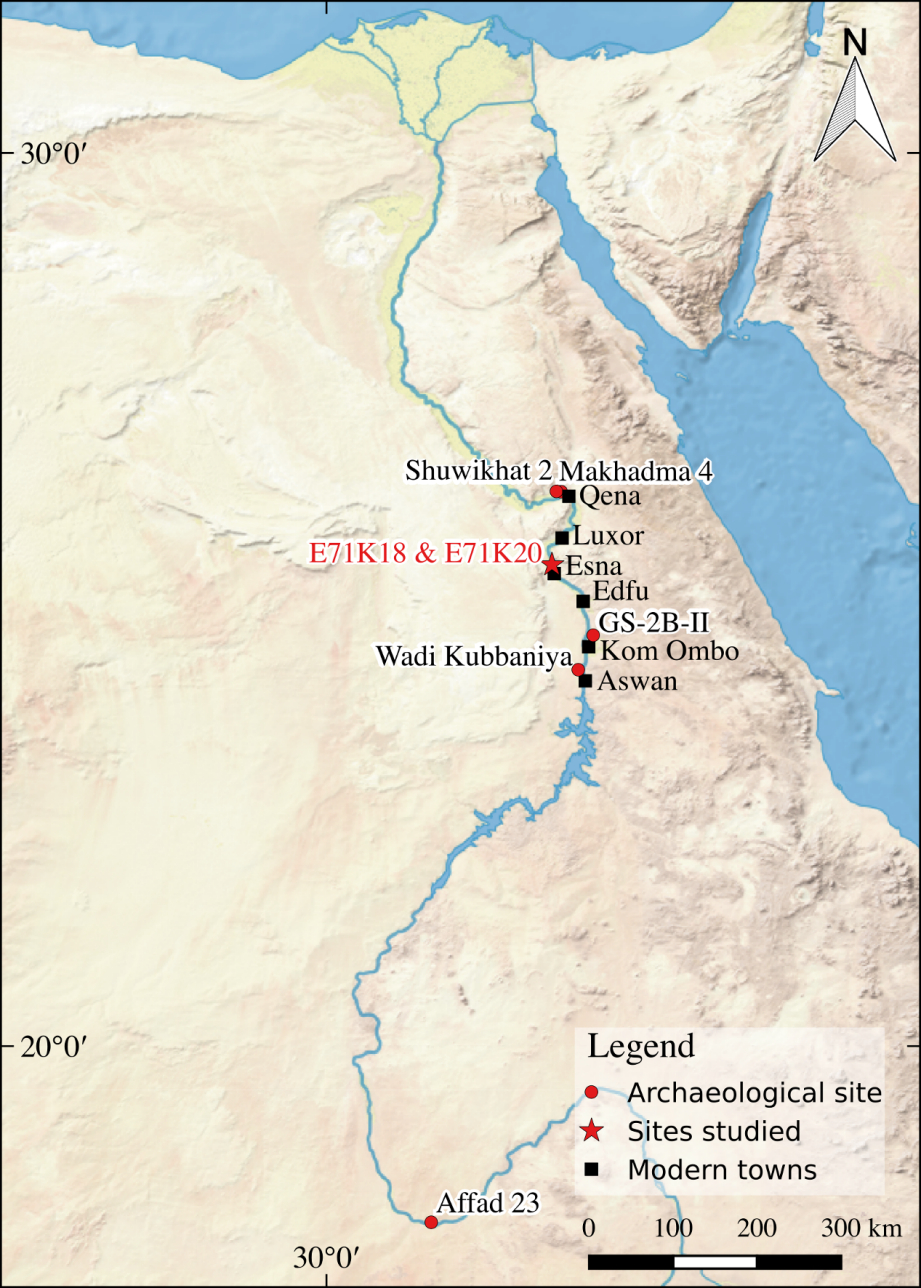
Location of the sites mentioned in the text. Created using Natural Earth Data in QGIS [33].

The Afian industry is known from E71K18 (type-site) and E71K6B in the Esna area. A small surface collection in Wadi Kubbaniya was also attributed to the Afian [31], as well as the site of Makhadma 4 in Middle-Egypt [21,22] and site GS-2B-I in Kom-Ombo ([24] refers to a personal communication by J. Phillips [26,32]) (Fig 1).

Site E71K18 was excavated and first described by Wendorf and Schild [24]. It is located near New Thomas Afia Village, a few kilometers north of Esna (Fig 1). The site comprises five distinct small and dense deflated surface concentrations (A to E) ranging from 10 to 20m in diameter. These concentrations are located close to a low escarpment of silts, within which a line of artefacts, charcoals and bone fragments could still be seen, indicating that part of these concentrations were still *in situ* [34]. Trenches were dug in four of the concentrations (A, B, C and E). The material was on a deflated surface associated with a pond sediment within a shallow swamp that dried up and refilled seasonally. This pond sediment is located above the silt and sand deposits associated with maximum aggradation and dune sedimentation in the area. Numerous fish and few mammal remains were associated with the cultural layers. All of the artefacts were collected and the top sediments were systematically sieved using a 1cm screen [34]. Occupation levels at the site are not dated. However, Schild and Wendorf [21] propose a date of around 16.5-15/14.5 cal ka BP for the Afian on the basis of stratigraphic correlations and lithic comparisons (but see discussion in 2.1.3). This takes into account the fact that other sites in the Esna area are associated with the lower deposits interpreted as the maximum aggradation, which have yielded 14C dates of around 17,000–18,000 BP (e.g. site E71K9X, I-3420, 16,830 ± 290 BP on a calcareous root cast [24]). In addition, the lithic artefacts from E71K18 present similarities with those from the site of Makhadma 4 (near Qena) and GS-2B-I (near Kom Ombo). Makhadma 4 has yielded seven radiocarbon dates on charcoal ranging from 12,940 ± 130 BP (GrN-12034) to 12,320 ± 100 BP (GrN-12981) [35], while GS-2B-I has three radiocarbon dates on *Unio* shells ranging from l3,560 ± l20 BP (Y-1447) to 13,240 ± 130 BP (SMU-123) [36,37].

The site presents an important lithic assemblage which suggests repeated or long-term occupation(s). The description of the Afian industry mostly relies on the lithic artefacts from this site [24,34]. The Afian industry is characterised by the production of bladelets and small elongated flakes from wide and flat opposed platform cores with facetted striking platforms [24]. The Levallois method is sometimes present. Retouched tools are mainly composed of atypical geometric microliths, truncated blades and flakes, and backed pieces. The microburin technique (MBT) was used to manufacture some of the truncations and geometrics. The Afian lithic assemblages at E71K18 and E71K6 show some variability in the frequencies of rounded opposed platform and single platform cores, the presence of Levallois cores (classical and Bent Levallois [38] cited in [34]) and the frequencies of retouched tool categories (geometrics, truncations and endscrapers). Close and colleagues [34] performed a stylistic study mainly based on typology in order to characterize the differences and similarities between the Afian assemblages. They concluded that all Afian assemblages are similar enough to justify their grouping into a single industry but that the analysis shows a tight cluster of sites 18A, 18C and 6B, with 18E being closely related, but 18B and 18D probably represent two other “bands” belonging to the same broader Afian group [34]. Here, I will consider concentration E71K18C.

Although Makhadma 4 has also been attributed to the Afian, it differs from E71K18 by the presence of numerous burins, arch backed pieces and bipointed bone artefacts while lacking Levallois and microburins. The Kom Ombo site GS-2B1 is characterised by the presence of grinding stones [21].

### The Silsilian industry and E71K20

The Silsilian industry was defined by Smith [25,39] as a non-Levallois microlithic industry including the use of the microburin technique associated with triangular and trapezoidal tool forms and the use of exotic raw materials. Based on this description, site GS-2B-II in the Kom Ombo plain (Fig 1) was attributed to the Silsilian because major features of its lithic assemblage are: microblade cores dominated by the single striking platform type, artefacts of microlithic dimensions, the presence of the microburin technique, and retouched tools dominated by truncations and backed bladelets [27]. Following this definition, the authors group GS-2B-II and E71K20B within the Silsilian, although “it is impossible to provide rigorous arguments for grouping GS-2B-II and E71K20B (…) the available qualitative descriptions leave little doubt that GS-2B-II and E71K20B fall within the basic parameters given by Smith for the Silsilian.” [27], p.367.

In the description of the assemblage from site E71K20, Wendorf and Schild [24] note that “it is very similar to the Silsilian but appears to be unlike any other known material from Upper Egypt”. The site is located in the Esna area south of Thomas Afia Village, about 5 km from E71K18. It is a surface site which comprises two distinct, dense concentrations (areas A and B), which were collected in totality. Burnt features were observed although they could not be related to the surface material. No archives with counts of the material were found in the British Museum apart from some notes. They indicate that the concentrations were about 10m apart from each other, and that E71K20A, cut by a road, corresponded to a 27x8.5m area, while E71K20B corresponded to a 20.8x16.2m area and may have been associated with a deflated hearth. Trenches were dug but no occupation layer could be found. On the basis of the similarities between the assemblage and site GS-2B-II in Kom Ombo, it was suggested that they could be contemporaneous, the latter site having yielded two radiocarbon dates, one on charcoal (15,310 +/- 200 BP (Y-1376)), the other on *Unio* shell (14,390 +/- 200 BC (I-5180)) [27], thus suggesting a date between 18.7 cal ka BP and 17.5 cal ka BP for the Silsilian and the occupation at E71K20 [21].

The Silsilian is similar to an industry defined in Lower Nubia, namely the Ballanan [40]. They were grouped into the Ballanan-Silsilian industry [21].

### Geomorphological context of the sites

Both sites were lying on sediments which were attributed to different formations (Ballana dunes for E71K20, also known as the Late Sahaba aggradation [41]). However, after Wendorf and Schild's research in Kubbaniya, all Late Palaeolithic sites from the Esna / Edfu region are now considered to be part of the “Late Palaeolithic alluviation”, of Late Pleistocene age according to the braided river model proposed by Schild and Wendorf [21,24,42] for the Late Pleistocene Nile. E71K18 would be located below the topmost silts of the Late Palaeolithic Alluviation at the time of a pond development. As for the site of E71K20, its age was suggested solely on the basis of its attribution to the Silsilian and similarities with the Kom-Ombo site GS-2B-II.

Recently, Vermeersch and colleagues [43] and Vermeersch and Van Neer [20] proposed a new model for the Late Pleistocene Nile whereby dunes from the Western Desert invaded the Nile Valley and formed dams, resulting in the creation of large lakes. In particular, they suggest that the geomorphology of the region of Esna (as described in the reconstruction of evolution of the landscape at Esna, fig 49 in [24]) “is even better understandable when the high Nile levels (…) are indeed Nile lake levels”. However, this model is debated: Schild and Wendorf [21] noted the absence of typical lake formations such as calcareous marls, diatomites or beach deposits. Vermeersch and Van Neer [20] responded that, contrary to the Wadi Kubbaniya lake (dammed by a wadi dune), “lakes in the Nile Valley were fed, even on an irregular basis, by Nile water, which is not favourable for such deposits” and that there is no evidence of important accumulation of sediments consistent with a braided river in the Nile Valley. If we accept Vermeersch and Van Neer’s suggestion that the sequence at Esna is better interpreted in terms of lake levels, then the relative altitude of the sites cannot be used as a relative chronology. Since there are no direct chronometric dates at either E71K18C or E71K20, their dating would rely only on techno-typological criteria. Furthermore, regardless of which interpretative model is chosen for Late Pleistocene Nile geomorphology, none of the radiocarbon dates used to discuss the chronological context of E71K18 and E71K20 are directly associated with these sites. Most of them were generated prior to the 1990’s, applied to samples stored sometimes for many years before being analysed, or derive from *Unio* shells which are susceptible to reservoir effects, particularly in the Eastern Sahara [44,45]. Given these limitations, the dating and/or geomorphological correlations of the occupations must be treated with caution.

## Research questions and methods

E71K20 was only briefly described and the description and analysis of E71K18 focused mainly on its stylistic and typological characteristics. The main research objectives of this paper are thus: (1) to describe and analyse a sample of these lithic assemblages from a technological point of view in order to highlight differences or similarities in the blank production and to relate it to typology; to (2) discuss variability between these industries; and (3) provide data which can be used in comparative studies with adjacent regions. With the exception of some assemblages which were left in Cairo at the Egyptian Department of Antiquities and some sent to the Polish Academy of Science in Warsaw, Poland [34], most of the “Wendorf collection” is stored at the Department of Ancient Egypt and Sudan in the British Museum in London, where data collection took place.

Permission to access the collections (E71K18C and E71K20A&B) was granted by the Department of Ancient Egypt and Sudan of the British Museum. Methods used for the analysis rely on the *chaîne opératoire* approach, combined with an attribute analysis in order to best characterise each assemblage (qualitatively and quantitatively) and compare between them. Within each assemblage, all artefacts were counted and assigned to broad techno-typological categories and subcategories (S1 Appendix): cores, core trimming elements (core tablets, crested products, debordant products (i.e. side core products), overpassed products, flakes with many elongated blank dorsal removals), elongated blanks (blanks with a length to width ratio ≥ 2), flakes ≥ 2cm, chips and flake fragments <2cm, microburins, retouched tools (truncations, backed pieces, notched pieces, geometrics, endscrapers, others). Both industries have been defined as blade(let) industries and the initial qualitative overview of the assemblages confirmed that both assemblages are oriented towards the production of elongated blanks. This first qualitative assessment of the assemblages allowed a stratified sampling strategy for the detailed analysis: in order to facilitate morphometric comparisons, only complete pieces within each of the subcategories defined above and related to elongated blank production were considered. Within each of the subcategories, complete pieces were randomly sampled so that the number of sampled pieces comprised at least 15% of the category and/or subcategory total. The attributes used in this analysis as well as the raw database can be found in S1 Appendix and S1 Database.

Elongated blanks (ratio length to width ≥ 2) were divided into large (blades) and small (bladelet) blanks, using a contextual approach based on a k-means cluster, following the protocol described in Pargeter and Redondo [46], based on the code written in R by C. and J. Pargeter [47]. Differences between assemblages were quantified using non-parametric statistical tests which allow analysis of small samples and variables that are not normally distributed, such as the Kruskal-Wallis test with the posthoc Wilcoxon and Fisher tests. Statistical analyses were conducted using R and RStudio [48,49] and graphs were produced using the ggplot2 package [50].

## The Afian lithic assemblage from E71K18C

### General characteristics

Preliminary observation of the lithic artefacts show that they are heavily wind-patinated, with a brownish color. The edges are often rounded. This suggests a long exposure to the surface which could have allowed the intrusion of artefacts within the assemblage.

Debitage products (Table 1) are composed of a relatively high frequency of elongated blanks (>22% of the assemblage, not counting chips). There is a very high frequency of retouched tools (23% of the assemblage, not counting chips). The retouched tools were the subject of a detailed typological and stylistic analysis by Close and colleagues [34].

**Table 1:** Count of all artefacts from E71K18C.

	N	%	% without chips and chunks	Sample studied*
**Cores**	**334**	1.8%	3.3%	**88**
**Flakes and flake fragments**	**3467**	19.1%	34.7%	**/**
**Elongated blanks and fragments**	**2240**	12.3%	22.4%	**314**
**including complete elongated blanks**	**709**	3.9%	7.1%	**314**
**primary flakes & blades**	**1743**	9.6%	17.4%	**/**
**core trimming elements**	**156**	0.9%	1.6%	**43**
**retouched tools**	**1674**	9.2%	16.7%	**380**
***Piquant-trièdres* (not further modified)**	**7**	<1%	<1%	**/**
**microburins**	**379**	2.0%	3.9%	**82**
**chips**	**7900**	43.5%		**/**
**chunks**	**273**	1.5%		**/**
**TOTAL**	**18173**		10000	**907**

* All pieces have been qualitatively evaluated using a limited set of variables. The sample comprises only the pieces for which all variables described in S1 Appendix have been recorded.

The material from E71K18C is divided into 33 bags, probably corresponding to the initial sorting of the material by A. Close and colleagues. A detailed inventory per bag is found in S1 Database, sheet a. The counts presented here vary slightly from the counts of A. Close and colleagues (p.36 in [34]): more core trimming elements were recognised, elongated blanks were separated from flakes, and slight differences occur in the count of retouched tools.

The raw material used is mainly chert, which probably comes from nearby outcrops of Nile or Wadi formations. All steps of stone tool production are present: primary flakes are well represented, as well as debitage, chips, cores and abandoned retouched tools (Table 1).

### Blank production

The main reduction sequence of the assemblage is oriented towards the production of elongated blanks, as indicated by both high frequencies of elongated blanks as well as cores with elongated negative scars (>75% of cores studied, Table 2).

**Table 2:** E71K18C –characteristics of cores (n = 88).

	Single platform cores	Opposed Platform Cores—same debitage surface				Two opposed platform cores—different debitage surfaces	Bent & Halfan	Multiple Platform Cores (incl. use of same debitage surfaces)	Mupltiple platform cores (use of distinct debitage surfaces)	TOTAL
* *	*Uni*.	*Bidir*.	*Uni*. *+ opp*.	*Uni*. *Succes*.	*indet*	*Uni*. *Success*		*Uni*. *Success*.	*Uni*. *Success*.	
**Globular**				1				1	1	**3**
**Oblong**	6	1		1				5		**13**
**Oval**			2	4			1			**7**
**Pyramidal**	5	1	1			2				**9**
**Quadrangular**	1	5	3	17	1	1		16	2	**46**
**Triangular**	5					1	4		** **	**10**
**TOTAL**	**17**	**7**	**6**	**23**	**1**	**4**	**5**	**22**	**3**	**88**
**%**	**19.3%**	**8.0%**	**6.8%**	**26.1%**	**1.1%**	**4.5%**	**5.7%**	**25.0%**	**3.4%**	

uni. = unidirectional, bidir. = bidirectional, opp. = opposed, success. = successive.

#### Geometric organisation of cores

Cores are mainly opposed platform (47%, n = 158/334) and multiple platform cores for producing flakes and blades (mostly with three or more orthogonal platforms, 16%, n = 52/334), 17% (n = 57/334) are single platform cores and 20% (n = 64/334) are unclassifiable or fragmentary. Eighty-eight cores have been analysed in this study (see Table 3). Apart from a few exceptions (N = 2/88 cores studied), cores display knapping surfaces with few convexities and the reduction almost systematically took place on the wider surfaces, indicating a planimetric conception of debitage (sensu [11]). Very few core trimming elements (CTE’s) are present in the whole E71K18C assemblage (<1%, Table 3), although more CTE’s were recognised during the present study than in the original count (N = 29, [34]).

**Table 3:** Types of core trimming elements at E71K18C.

	N	%
**Overpassed products**	68	43.6%
**Crested blanks**	5	3.2%
**Platform blades**	12	7.7%
**Core tablets**	15	9.6%
**Products with elongated scar removals**	9	5.8%
**Debordant products**	28	17.9%
**Other convexities management products**	19	12.2%
**Total**	**156**	

The miscellaneous convexity management products may be associated either with flake or elongated blank production, but all other core trimming elements (>85%) are related to elongated blank production. They are dominated by overpassed products (44%), with half of them (N = 32) removing part of a distal striking platform which is consistent with the high percentage of opposed platform cores.

There are no significant differences in the dimensions of cores according to their types (single platform, opposed platform or multiple platform cores). The length of most cores ranges from 34 to 43mm (mean length = 38.6mm, sd = 7.3). However, differences in length and thickness are observed between cores for flakes, cores for both flakes and elongated blanks, and cores for elongated blanks only (S1 Table, sheet a). Flake cores are shorter, while cores with elongated scar removals are longer, more elongated and comparatively more robust, with a lower width to thickness ratio. This is especially the case when compared to cores with both flakes and elongated blank removals. Since no technological characteristics from the cores allow one to distinguish flake production from elongated blade production, these results would be consistent with a continuity between elongated blank and flake production, the latter occurring during the last phase of core reduction.

**Fig 2:**
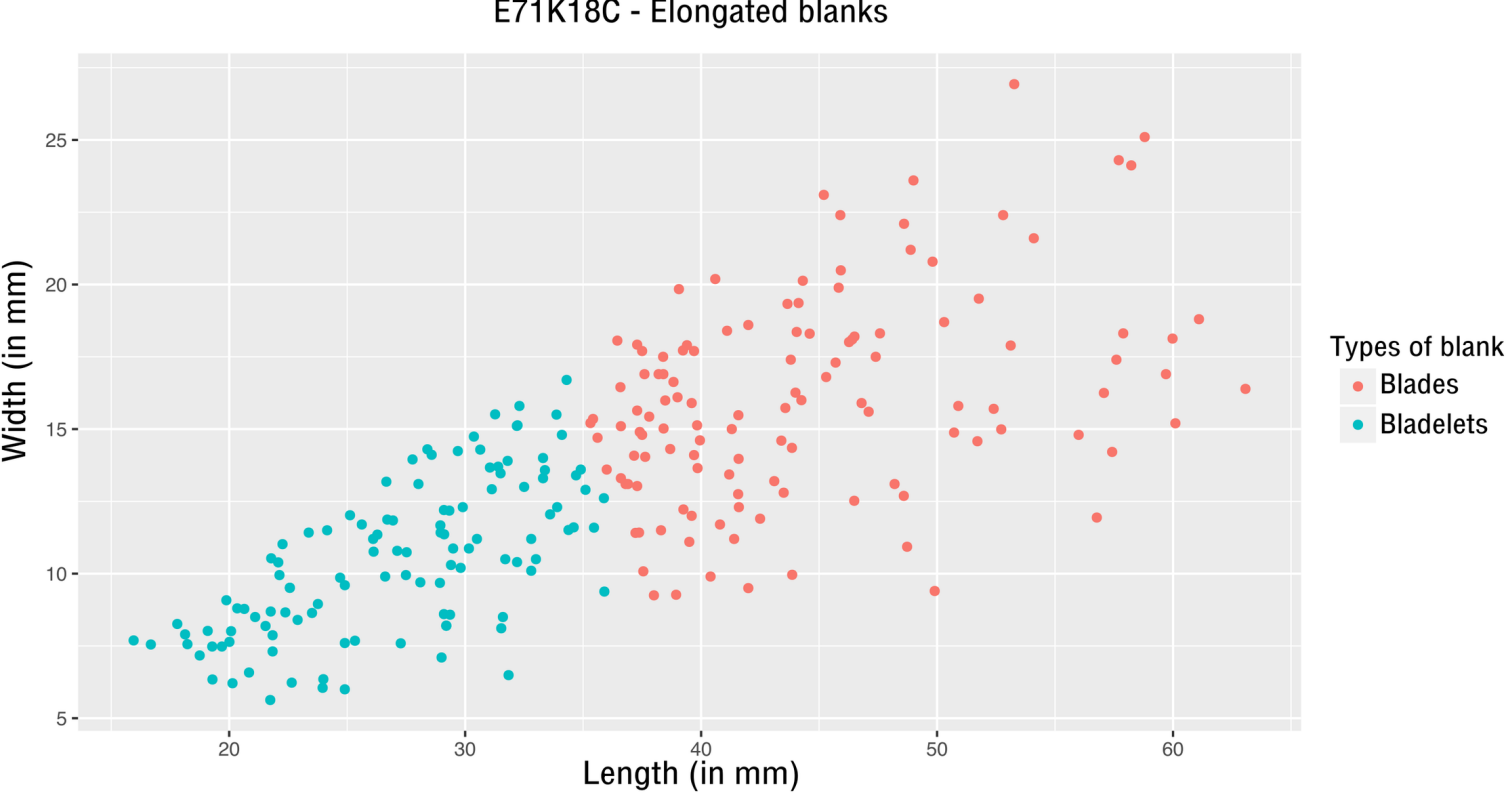
E71K18C – plot of length and width of elongated blanks.

Complete elongated blanks (N = 236) are relatively short and wide (mean length = 35.8mm, sd = 10.6) with a length to width ratio of 2.8 (sd = 0.6)). The results of a k-means cluster analysis allow the distinction of smaller blanks (bladelets, mean length = 27.2mm, sd = 5.1) from larger blanks (blades, mean length = 44.4mm, sd = 7) (S1 Table, sheet b and Fig 2). However, this metric distinction between blades and bladelets is not reflected in the cores and does not seem to correspond to technological differences (see below).

#### Direction of core reduction, platform management and dorsal surface convexity

With the exception of a few cores displaying intercalated negative scars from opposed striking platforms, two-opposed and four-orthogonal platform cores usually show the exploitation of the knapping surface(s) from each platform one after the other, with the direction of *debitage* remaining mainly unidirectional. In addition, most of these cores have a quadrangular shape which may be a logical consequence of the successive use of opposed striking platforms. This is consistent with the scar patterning observed on the dorsal surface of blanks, mainly unidirectional (63% of elongated blanks) and bidirectional (including unidirectional with few opposed removal scars) (20% of elongated blanks). The relatively high frequency of bidirectional scar removals may indicate a higher frequency of “true” bidirectional debitage, or unidirectional with maintenance of convexities from the opposed platform, than suggested from the data on cores. Since cores represent the last stage of reduction, this could be explained by bidirectional knapping taking place during the early stages of the reduction sequences. Results of the Kruskal-Wallis and Wilcoxon posthoc tests show that there is indeed a significant difference (Kruskal-Wallis chi-squared = 24, p-value <0.05) between elongated blanks with a bidirectional scar pattern (mean length = 41.3mm, sd = 7.9) and elongated blanks with a unidirectional scar pattern (mean length = 33.9mm, sd = 10.5).

Plain (43%) and dihedral or faceted (54%) striking platforms dominate the cores (n = 177 striking platforms for 88 cores). This is consistent with the data from elongated blanks, which show mainly plain (39%), or dihedral or faceted (39%), and linear or punctiform (21%) platforms. The high percentage of faceted butts denotes a careful preparation of the striking platform before the removal of the elongated blanks, consistent with the cores’ characteristics.

All blanks present wide butts and most of them show prominent bulbs (S1 Table, sheet c). The most likely hypothesis here is the use of direct percussion with a hard hammerstone [51,52]. The only difference in proximal characteristics between small (bladelets) and large (blades) elongated blanks is the relative percentages of faceted and linear/punctiform butts, which may be a consequence of the size of the blanks (S1 Table, sheet c).

Most of the blanks have distally convergent (40%) or parallel edges (34%), and show a slightly curved to curved longitudinal profile (71% of elongated blanks) with a flat lateral profile (86% of elongated blanks).

The lithic assemblage from E71K18C is oriented towards the production of elongated blanks. Although two clusters corresponding respectively to smaller and larger elongated blanks can be differentiated based on the length and width of the products, they do not seem to correspond to two distinct technological groups. These two clusters may instead correspond to a bias related to the size of the raw material, creating two “artificial” clusters.

### Levallois flaking systems

**Fig 3:**
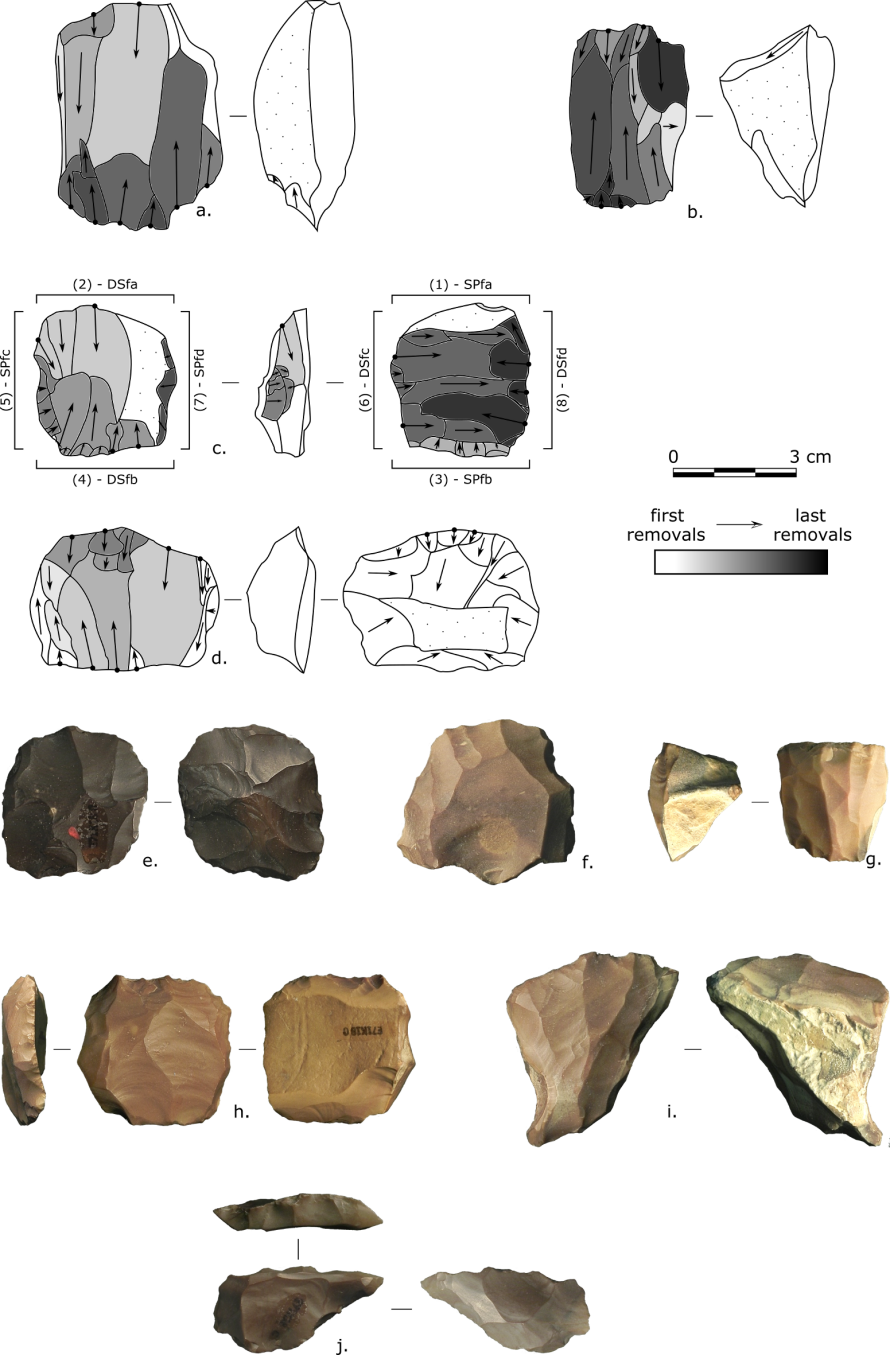
E71K18C – cores. (a,b) Opposed platform cores; (c-e) Multiple platform cores; (f) Halfan core; (g-i) Single platform cores, (h) retouched; (j) “Bent Levallois core”. Photos and drawings: Alice Leplongeon, taken courtesy of the Trustees of the British Museum.

A particular category of artefacts, sometimes called “Bent Levallois cores” [34,38], was also observed. They present a characteristic triangular offset shape with an abrupt straight distal end which may present the form of a retouched edge. One of these objects does not show any struck removal. These artefacts may have been primarily intended as bifacial tools and then later served as opportunistic cores (Fig 3J). Six products can be related to the Bent Levallois cores: they present a *déjeté* triangular form with a centripetal scar pattern. Numerous Bent Levallois cores are present at another site attributed to the Afian: E71K6B [34].

One additional core presents Levallois characteristics consistent with the definition of the Halfan method, a form of Preferential Levallois technology with distal preparation taking the form of parallel elongated removals [53]. Seven other products are of Levallois-type; however, because the assemblage comes from a surface collection, their presence is difficult to interpret.

### Retouched tools

Retouched tools are numerous in the assemblage (over 15% of the assemblage, excluding chips; see Tables 1 and 4). The dominant categories are truncations, backed bladelets and geometric microliths (35%, 20% and 10% of retouched tools respectively). A detailed typological analysis of the retouched tools was made by Close and colleagues [34], following Tixier's typology [54]. Here, a representative sample of the most common retouched tools is described and analysed using attribute analysis to allow further comparisons.

**Table 4:** E71K18C – retouched tools.

	Count (this study)		sample studied
	N	*%*	N
*endscrapers*	161	*8*.*98%*	45
*backed bladelets*	349	*19*.*48%*	82
*notched and denticulated pieces*	90	*5*.*02%*	24
*truncations*	622	*34*.*71%*	126
*geometric microliths*	174	*9*.*71%*	87
*retouched elongated blanks*	198	*11*.*05%*	16
*retouched flakes and varia*	198	* *	/
**Total**	**1792**		**380 (21%)**

### Main characteristics

Given the nature of the retouched tools, a high frequency of the types of blanks used for retouch are indeterminate (47%), especially for geometrics (82%). From those which could be determined, elongated blanks were clearly the preferred blank for retouch (44%).

**Fig 4:**
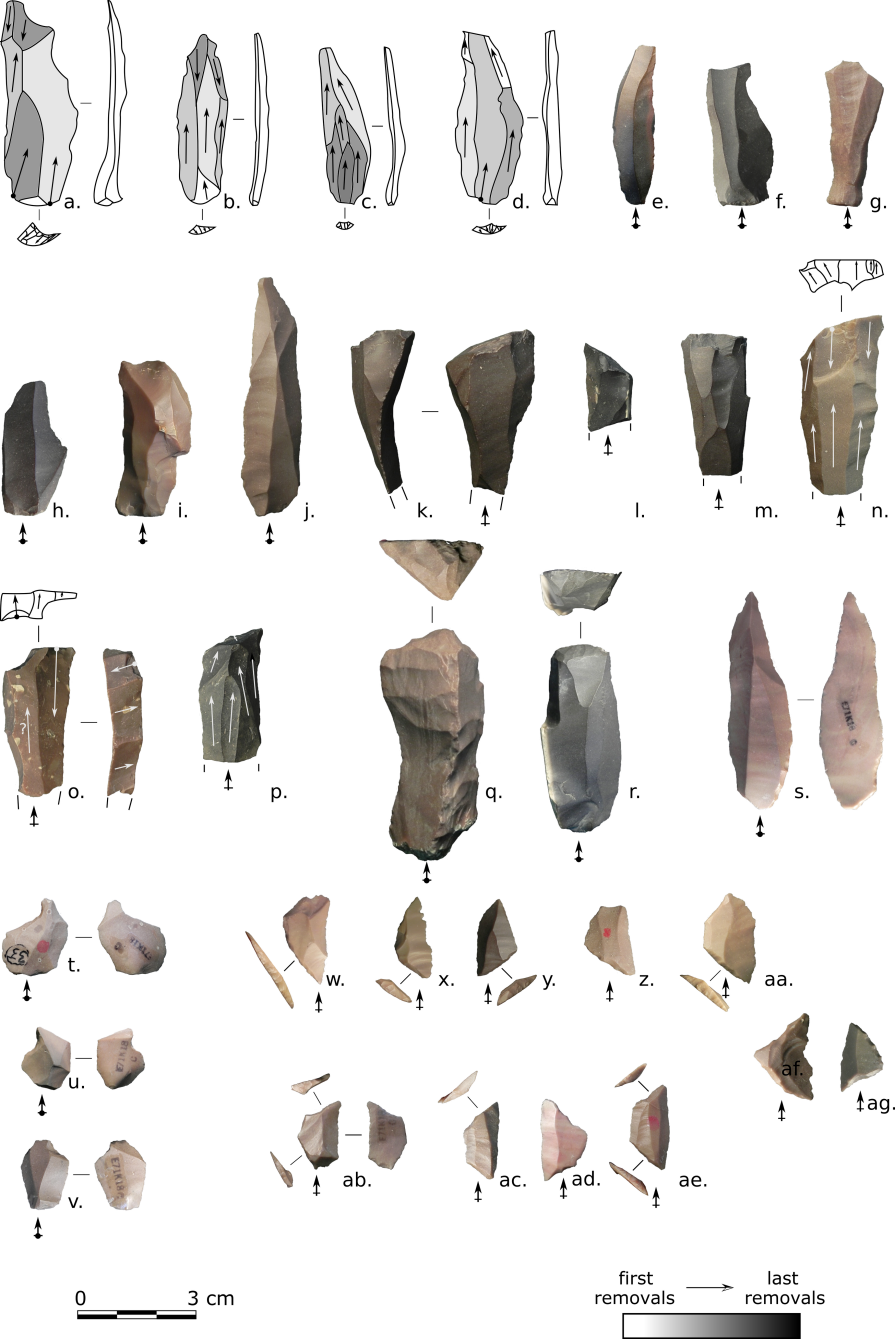
E71K18C – blanks and retouched tools. (a-j) Elongated blanks; (k-p) Overpassed blanks; (q,r) Endscrapers, (q) on a core trimming element; (s) Proximally retouched blade; (t-v) Proximal microburins; (w-aa) Truncations; (ab-ag) Geometrics (Trapezes and triangles). Photos and drawings: Alice Leplongeon, taken courtesy of the Trustees of the British Museum.

**Fig 5:**
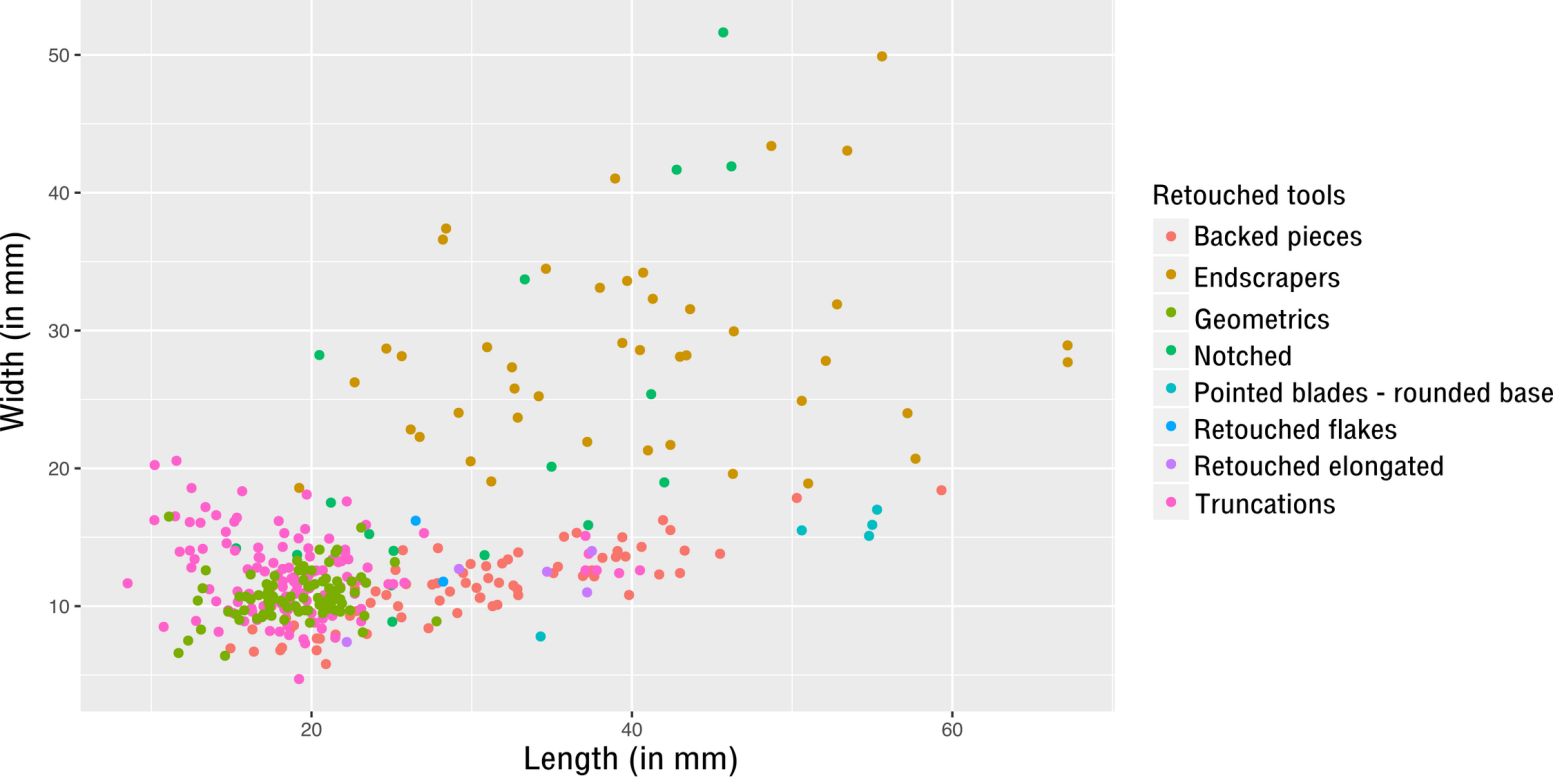
E71K18C—Plot of length and width of complete retouched tools.

Dimensions of the sample studied are consistent with results published by Close and colleagues (appendix 4 in [34]). Endscrapers are the largest tools (mean length around 40mm, s.d. = 11.7) followed by retouched pieces (with highly variable dimensions) and backed pieces (mean length around 30mm, s.d. = 8.8; see S1 Table, sheet d and Fig 5). Endscrapers are mainly made on primary flakes or core trimming elements (Fig 4) which denotes the absence of specific blank production for the manufacture of endscrapers: their manufacture is embedded within operational schemes primarily aimed at the production of other types of tools.

With the exception of a small group of larger truncations, the dimensions of geometrics and truncations overlap (mean length around 19mm, s.d. = 3.1 and 5.6 respectively; mean width around 11-12mm, s.d. = 1.7 and 2.8 respectively). They form a distinct microlithic group (Fig 5). Despite their rather tight clustering, the coefficient of variation (CV) (defined as the standard deviation divided by the mean and multiplied by 100) of both truncations and geometrics is too high to indicate real standardisation (see S1 Table, sheet d and see [55] for the choice of the limit at 10% for the CV to indicate standardisation).

Truncations are the most numerous retouched tools in E71K18C (see Table 4). Most of them are oblique proximal truncations (N = 125/126). Only three show the typical scar of a *piquant-trièdre*, which would indicate an occasional use of the microburin technique (MBT). The others show direct abrupt retouch.

Geometrics represent over 10% of all retouched tools (Table 4). Most are trapezes and triangles, with only a very small number of lunates observed (Table 5 and [34]). Trapezes and triangles are preferentially retouched on the left edge which has been interpreted as an expression of style [34]. Trapezes and triangles group together a variety of subtypes (see Table 5 and [34]), but for comparative purposes they will be considered here as two broad categories (triangles and trapezes). Some triangles and trapezes are made on *piquant-trièdre* (see Table 5). Trapezes and triangles do not show any major differences in their dimensions (see Table 5 and Fig 4). Each category shows a low level of standardization; the coefficients of variation of their dimensions are above 10% (see Table 5).

**Table 5:** E71K18C: Types of geometrics according to close et al. [34] and sample studied.

	Close et al. [34], p. 157 and p. 180		Sample studied
	N	*%*	N
**lunates**	**9**	***4*.*5%***	**/**
**trapezes**	**112**	***55*.*7%***	**41**
incl. Sinister	85		28
*with MB scar prox*	*8*		*2*
*with MB scar dist*	*25*		*6*
incl. Dexter	26		13
*with MB scar prox*	*9*		*4*
*with MB scar dist*	*1*		*0*
**triangles**	**80**	***39*.*8%***	**46**
incl. Sinister	51		31
*with MB scar prox*	*14*		*2*
*with MB scar dist*	*19*		*2*
incl. Dexter	22		14
*with MB scar prox*	*13*		*3*
*with MB scar dist*	*1*		*0*
**Total**	**201**	** **	**87**

Backed pieces are the second most frequent retouched tools in the assemblage. Although they are small (all but one are less than 50mm long, with a mean length around 30mm), they form a distinct group from the other microlithic tools (truncations and geometrics, see Fig 5). From the sample studied (N = 72), all were made on elongated blanks with dimensions corresponding to bladelets and small blades. Most have their proximal portion (butt and bulb) removed (N = 59/82, 72%). Retouch is abrupt and often localised on the proximal part of the left edge. The resulting shapes are quite diverse with little standardisation, ranging from pointed or rounded bases with convergent edges, to generally divergent edges with a pointed tip, to almost crescent-like shapes.

#### Use of the microburin technique (MBT)

Two microburin indexes, defined by Henry [56], may be used to evaluate the significance of the microburin technique within an assemblage: the microburin index (Imbt: ((nb of microburins)/(nb of microburins + nb of retouched tools)) * 100) and the restricted microburin index (ImbtR: ((*nb of microburins*)/(*nb of microburins* + *nb of backed tools*)) * 100). A threshold of 50 for the ImbtR is used to indicate a habitual and intensive use of the MBT. At E71K18C, the Imbt is 15.1 while the ImbtR is 26.4 [34]. This low restricted microburin index probably reflects an occasional use of the microburin technique to manufacture geometrics and truncations.

**Fig 6:**
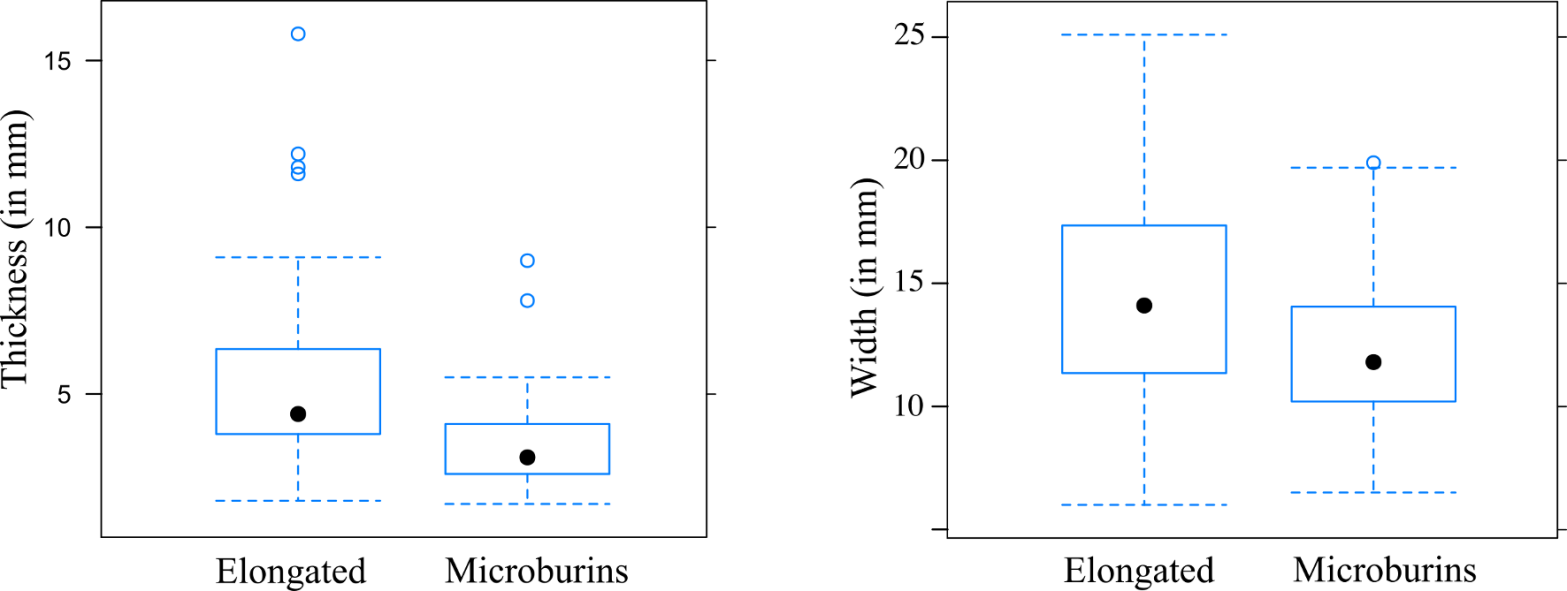
E71K18C – box plots of width and thickness of microburins compared with elongated blanks.

Eighty-two pieces related to the microburin technique were sampled (out of 386, see Table 1). They are mainly left proximal microburins (N = 41/82), some being mis-struck pieces with the microburin scar on the ventral face for the *piquant-trièdre*, or the reverse for the microburins (N = 10/82). In addition, a few *piquant-trièdres* were found without further modification. The microburins show no standardisation in their dimensions. Their width and thickness are within the range of the small elongated products which were likely selected for use of the microburin technique (see S1 Table, sheet e and Fig 6). The range of microburin angles is very large, showing little standardisation in the technique itself (see Fig 4). This is similar to the range of truncation angles measured on geometric microliths. These characteristics, associated with the relatively high number of mis-struck microburins, would reinforce the hypothesis that the microburin technique was only occasionally used. It may have been used only for a specific type of tools, such as geometrics since around 45% of geometrics appear to have been made on *piquant-trièdres* [34] (Table 5). In addition, the production of a high frequency of backed pieces could have led to the production of non-intentional microburins.

### Summary of E71K18C

The attributes analysed for cores, blanks and core trimming elements indicate that production is oriented towards making wide and small elongated products, using a hard hammerstone, following a planimetric conception of debitage. The striking platforms are often carefully faceted in order to produce these blanks. The flaking surfaces are usually not prepared, relatively convex (as indicated by the longitudinal profiles of the blanks), short and wide (with the cores often showing a quadrangular shape and being wider than long). The flaking mainly relied on the principle of recurrence, from one single or two opposed striking platforms, either used successively, or one of them used for the maintenance of convexities. Occasionally, a core tablet was used to rejuvenate the striking platform. In the case of opposed striking platforms, overpassed blades were produced, which may be considered as knapping “errors” or deliberate by-products to maintain distal convexities. Cores were heavily reduced which is seen in their small dimensions, often smaller than the elongated blanks.

Blanks were transformed into a variety of retouched tools: mostly proximal truncations, backed bladelets and geometrics, with a high frequency of notched and denticulate tools, as well as some endscrapers. The microburin technique was occasionally used for the manufacture of truncations and geometrics.

## The Silsilian lithic assemblages from E71K20

Despite similarities between the assemblages, differences in their composition were noted: E71K20A, grouped in three bags, lacks retouched pieces and microburins, and includes few core trimming elements. E71K20B, grouped in ten bags, includes many retouched pieces, core trimming elements and all other artefact categories. The field notes, stored at the British Museum, mentioned that the two concentrations are part of the same site. A representative sample of both concentrations was analysed for this paper (Table 6).

**Table 6:** E71K20 – counts of lithic artefacts.

	N—E71K20A	N—E71K20B	N—E71K20	%	% without chips and chunks	Sample studied*
**Cores**	24	124	148	2.0%	2.5%	60
**Flakes and flake fragments**	710	1386	2096	29.0%	35.2%	/
**Elongated blanks and fragments**	453	1584	2037	28.1%	34.2%	215
*including complete elongated blanks*	*86*	*374*	*460*	*6*.*4%*	*7*.*7%*	*181*
**primary flakes & blades**	305	688	993	13.7%	16.7%	/
**core trimming elements**	47	197	244	3.4%	4.1%	108
**retouched tools**	2	393	395	5.5%	6.6%	118
***Piquant-trièdres***		2	2	<1%	<1%	
**microburins**	1	45	46	0.6%	0.8%	20
**chips**	106	999	1105	15.3%		/
**chunks**	/	175	175	2.4%		/
**TOTAL**	**1648**	**5593**	**7241**	** **	**5987**	**521**

* All pieces have been qualitatively evaluated using a limited set of variables. The sample comprises only the pieces for which all variables described in S1 Appendix have been recorded.

### Blank production

Although dominated by flakes, the assemblages are clearly oriented towards elongated blank production, comprising almost a third of the assemblages.

#### Geometric organisation of cores and dimensions

**Fig 7:**
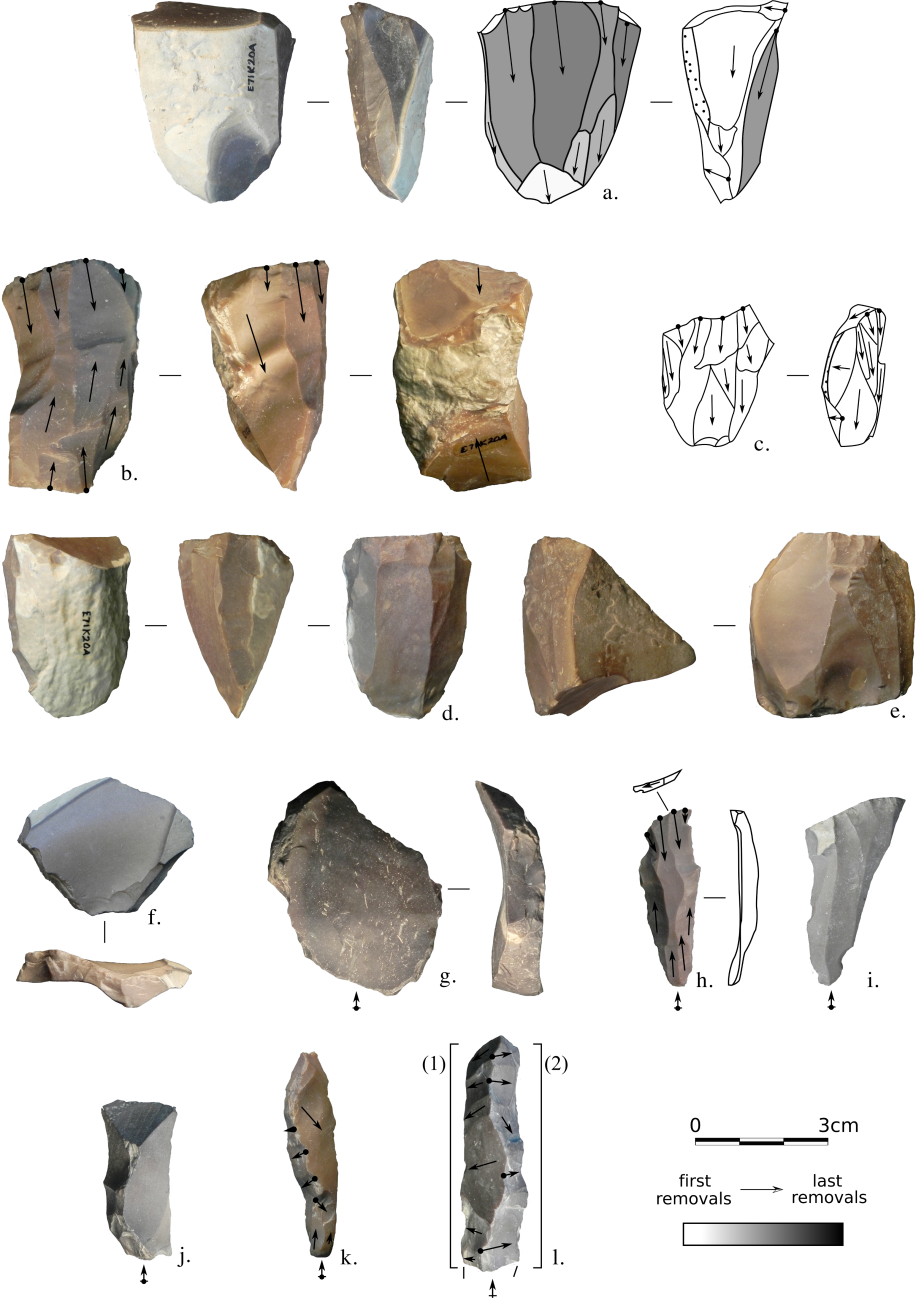
E71K20 – cores and core trimming elements. (a,c-d): single platform cores; (b,e): opposed platform cores; (f-g): core tables, (h,i): overpassed blades, (h) with opposed striking platform; (j-l): ridge products. Photos and drawings: Alice Leplongeon, taken courtesy of the Trustees of the British Museum.

Cores are dominated by opposed and single platform cores for the production of elongated blanks (46% and 25% respectively; see S2 Table, sheet a and Fig 7). In the sample studied, only one single platform core is on a flake, the others being on cobbles. Cores are usually oblong (33%), quadrangular (27%) or pyramidal (17%) in shape (Table 7).

**Table 7:** E71K20 – characteristics of the cores.

	Globular	Oblong	Pyramidal	Quadrang	Quadrang-Pyr	Triangular	Total
**Single platform cores**			8	2	5	2	**17**
*undirectional*			8	1	2	2	13
*unidirectional with possible previous opposed platform*				1	3		4
**Opposed platform cores**		17		11	1		**29**
*unidirectional successive*		6		6			12
*bidirectional*		9		4			13
*unidirectional with few opposed removal scars (maintenance*?*)*		2		1	1		4
**Two offset platform cores**	2	3	1		1		**7**
*unidirectional successive*	2	3	1		1		7
**Two platforms, two debitage surface cores**	2		1				**3**
*unidirectional successive*	2		1				3
**Multiple platform cores**	1			1			**2**
*unidirectional successive*	1			1			2
**Others (indet)**	2						**2**

Core trimming elements are numerous (see Table 8 and Fig 6). With the exception of the miscellaneous core trimming elements which could correspond to either flake or elongated blank production, all others are likely related to elongated blank production and are consistent with a volumetric conception of debitage: crested products (37%), overpassed products (22%, including N = 29/53 which removed part of a distal platform), debordant products (9%), and core tablets and platform blades (9%).

**Table 8:** E71K20 – core trimming elements.

	N	%
**Overpassed products**	53	21.5%
**Crested blanks**	92	37.4%
**Platform blades**	6	2.4%
**Core tablets**	16	6.5%
**Products with elongated scar removals**	1	0.4%
**Debordant products**	23	9.3%
**Other convexities management products**	55	22.4%
**Total**	**246**	

In addition to ridge blades, aiming at controlling the longitudinal convexities of the cores, a number of single crested products may represent redirecting products. Instead of corresponding to a longitudinal core removal, they rather aimed to remove part of a former striking platform and the proximal parts of elongated blanks (lateral core removal), leading to the creation of a new striking platform at a slightly different angle.

**Fig 8:**
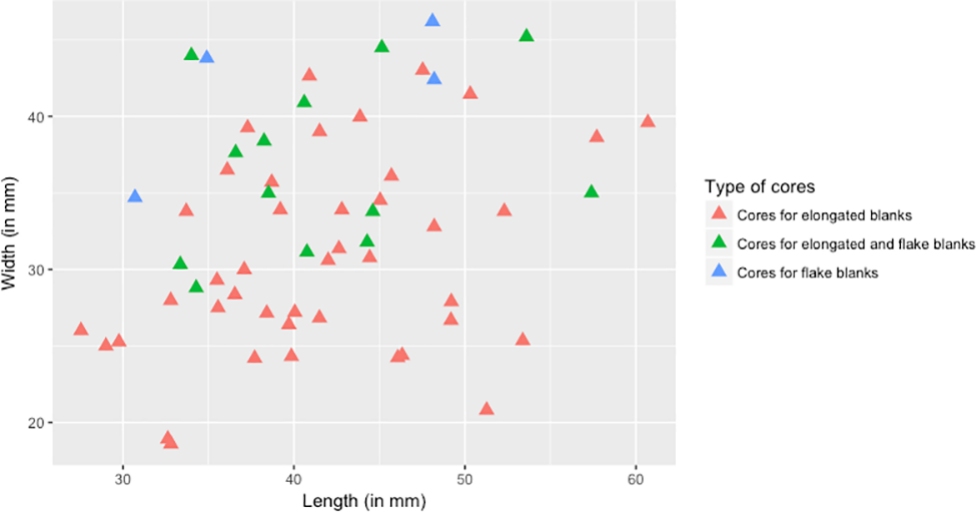
E71K20 – dimensions of cores.

Cores are small, ranging from 27 to 61mm long (mean length = 41.6mm, sd = 7.5; Fig 8). They do not show any clustering according to the type of cores (i.e. single vs opposed vs multiple platform cores). However, cores for elongated blanks are significantly narrower (mean width = 30.7mm, sd = 6.4) and therefore more elongated than the other cores (S2 Table, sheet b). It may indicate that elongated blanks come from several types of cores, including one with an elongated shape.

Complete elongated blanks are relatively short (mean length = 39.8mm, sd = 10.1) and wide (mean length to width ratio = 2.8 (sd = 0.6)). A k-means cluster analysis allowed the distinction between bladelets (mean length = 32mm (sd = 5.8)) and blades (mean length = 48.1mm (sd = 6.5)), see S2 Table, sheet c.

#### Direction of core reduction, platform management and dorsal surface convexity

Analysis of the scar pattern from the cores’ debitage surfaces shows that more than half of the cores present a unidirectional or unidirectional successive (when more than one striking platform) direction of core reduction, while 22% present a bidirectional scar pattern, and 13% display an opposed platform used for maintenance of distal convexities (Table 7). Blanks with unidirectional, and unidirectional and lateral scar patterns (in relation to the presence of ridge blades) are therefore expected to dominate, as well as a high percentage of blanks with bidirectional or unidirectional scar pattern with a few opposed scar removals. Data from elongated blanks are consistent with this model, showing a dominance of unidirectional (47%) and unidirectional and lateral (20%) scar pattern, as well as bidirectional (18%) and unidirectional and opposed scar pattern (9%). The emphasis on the preparation of the debitage surface, suggested by the high number of core trimming elements, may also be seen in the high number of dorsal scars (mean of 4.5 for the elongated products).

There are no significant differences in the dimensions of the blanks according to their scar pattern (Kruskal-Wallis p-value >0.05). Two principal different core reduction strategies for the production of elongated blanks can be suggested: from single platform cores using unidirectional knapping, or from opposed platform cores using bidirectional knapping.

Most of the cores show plain striking platforms (N = 97/101 striking platforms). Similarly, 62% of elongated blanks have a plain butt and 29% a linear or punctiform butt. Butts are relatively narrow (mean butt breadth of 5.7mm (sd = 2.8) and thin (mean butt thickness of 2.4mm (sd = 1.5)) (see S2 Table, sheet d). These butts are usually associated with a prominent bulb (around 50% elongated products) and the edge of the butt was abraded for 74 elongated blanks (~40%). These characteristics suggest the use of a soft stone hammer.

Most of the blanks show a slightly curved (35%, n = 64/186) to curved longitudinal profile (33%, n = 65/186) which indicates that the debitage surfaces were relatively convex. The high frequency of flat lateral profile (93%) shows that the lateral convexities were well maintained. Most of the blanks have rounded edges (33%) or convergent or distally convergent edges (43%), with few truly parallel edges (15%) observed.

### Retouched tools

**Fig 9:**
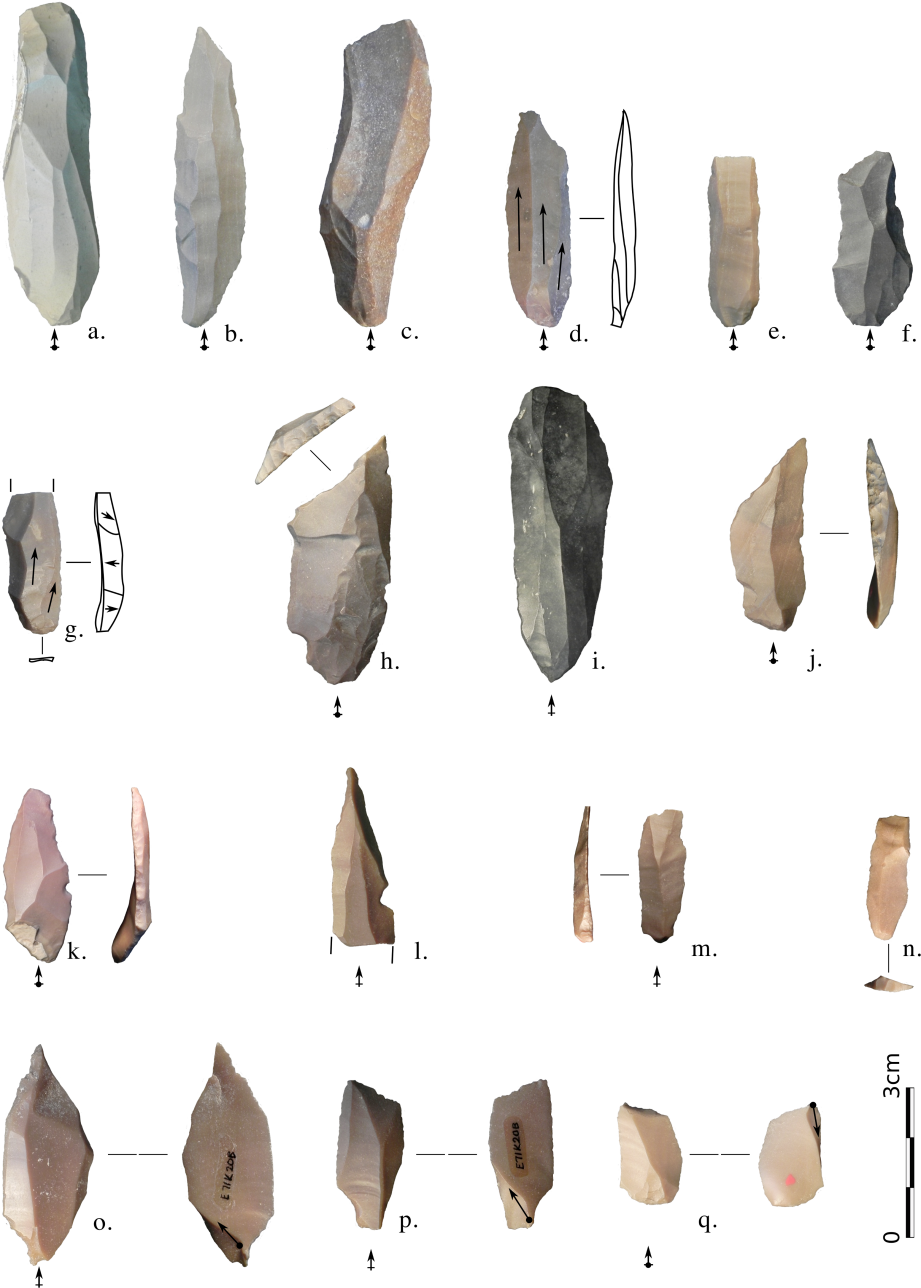
E71K20 – elongated blanks and retouched tools. (a-g): elongated blanks; (h): distal truncation, (i): blade with an ogival retouched base, (j,k): backed blades, (l): perforator, (m,n): proximally retouched bladelets, (o-q): microburins, (o,p), distal, (q) proximal. Photos and drawings: Alice Leplongeon, taken courtesy of the Trustees of the British Museum.

A total of 395 retouched tools were found in the assemblages (see Table 9 and Fig 9). The most numerous tool type are backed pieces (>55%) and, in particular, distally backed pieces (25% of total retouched tools) which consist of oblique truncations of elongated blanks, usually forming a point.

**Table 9:** E71K20: Retouched tools.

	Count (this study)		sample studied
	N (N complete pieces)	*%*	N
*Proximally backed pieces*	28 (17)	*7*.*1%*	14
*Distally backed pieces*	97 (34)	*24*.*6%*	45
*Arch backed pieces*	29 (29)	*7*.*3%*	15
*Partially backed pieces*	2 (2)	*0*.*5%*	1
*Backed fragments*, *miscellaneous*	67	*17*.*0%*	
*Double proximal retouch*, *elongated*	41 (18)	*10*.*4%*	18
*Ouchtata*, *elongated*	16 (5)	*4*.*1%*	1
*Notched*, *elongated*	28 (9)	*7*.*1%*	16
*Endscrapers*	5 (5)	*1*.*3%*	
*Burins*	6 (6)	*1*.*5%*	
*Other retouched*, *elongated*	33 (10)	*8*.*4%*	4
*Other retouched*, *flakes*	43 (37)	*10*.*9%*	
**Total**	**395 (172)**		**114 (77)**

**Fig 10:**
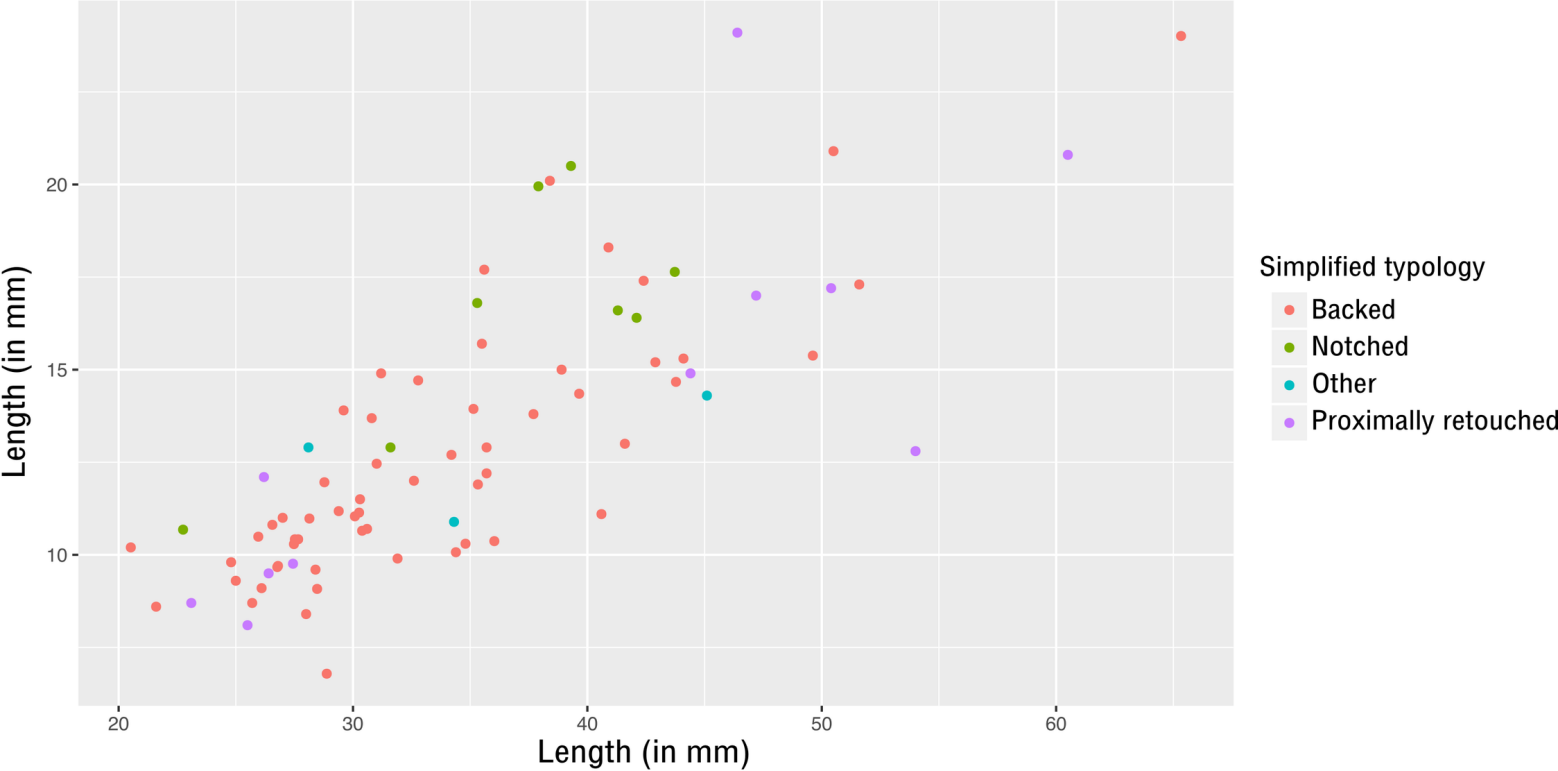
E71K20—Plot of length and width of complete retouched tools.

Most of the retouched tools are made on elongated blanks without any cortical surface (~85%). Complete retouched tools from the sample (N = 77) show that small elongated blanks predominate (<40mm long and <15mm wide, see Fig 10). There is no significant difference in size between tool categories (S2 Table, sheet e), although backed pieces tend to be smaller (mean length = 33.6mm (sd = 8.1)) than other tools. Proximally retouched pieces (mean length = 39.2mm (sd = 13.6)) may be divided into two groups based on size: those made on bladelets (<30mm long) and those made on blades (>40mm long) (Fig 10).

Among the backed pieces, distally truncated pieces dominate retouched tools (N = 97 compared to N = 38 proximally backed pieces). More than half of the studied truncated backed pieces (58%) have an angle of less than 55°, creating an acute distal (or proximal) pointed tip. Most of the distally or proximally backed pieces were made using relatively thick (between 2 and 4mm) abrupt direct retouch. Seven pieces present possible traces of the microburin technique with remnants of a small distal notch. The length of the retouch varies from 7mm to more than 27mm long. Arch backed pieces display a variety of types: for half of them, the backed retouch led to the removal of the butt and bulb of percussion, while many (N = 6/17) have the proximal part of the opposite edge of the back also retouched.

Proximally retouched blade(let)s are the second most common retouched tool type at E71K20. The retouch is direct, almost always abrupt, and is located on the proximal part of the blank, usually on both edges. It often leads to the removal of the butt and bulb of percussion (n = 12/18), but this is not systematic. The primary technical aim of the retouch is therefore not thinning of the proximal portion since the remaining bulbs are prominent. The general morphology of the tools is rounded (N = 10/18), divergent (N = 4/18), or divergent to parallel (N = 3/18).

Notched pieces on elongated blanks are the third most common group of retouched tools at E71K20 (7% of retouched tools). On most pieces, the notch is located on the mesial part of the edge (N = 8/16), though proximal and distal notches also occur (respectively N = 5 and N = 2). One of the notched pieces has abrupt retouch on the edge opposite to the notch. Although it is possible that those notches were related to the microburin technique, the depth of the notches and the presence of one notch with an opposite backed edge would more likely indicate that they are notched tools.

Whilst backed pieces clearly dominate the retouched tools, there is no clear homogeneity in their type, shape or dimensions.

### Use of the microburin technique

Out of the 36 pieces labelled “microburins” that E71K20B yielded, 20 are considered here; the others did not show the hinged fracture characteristic of microburins according to their strict definition [54]. The microburin index (Imbt) is 9 and the restricted microburin index (ImbtR) is 12. They are mainly distal microburins (N = 14) and show remnants of the notch on the left side, while the notch on proximal microburins is on the right side. Among the tools, only distal truncations showed traces of microburin blows which would be consistent with an occasional use of the microburin technique for manufacturing truncations.

They show a wide range of angles of the microburin blow, from 19 to 75° (mean around 47°), showing low standardisation. Microburins have variable dimensions; however, when their range of width and thickness is compared with dimensions for elongated blanks, notched pieces, and truncations, microburins correspond to truncations and the lower range of elongated products. This is consistent with a use of the microburin technique on the smaller elongated blanks.

### Summary of the assemblage from E71K20

The analysis of cores, blanks and core trimming elements indicate that core reduction at E71K20 is mainly oriented towards the production of short elongated products with a slightly curved to curved longitudinal profile and mainly rounded or distally convergent edges. The striking platforms were usually simply prepared (by a single blow) leading to plain butts. The convexities of the debitage surface were carefully maintained using ridge blades, debordant and overpassed products (high frequency of CTE's). Rejuvenation of striking platforms was done through the removal of core tablets.

Elongated blanks were transformed into retouched tools, mainly composed of backed pieces, particularly distally truncated pieces with backed retouch forming a point, proximally retouched blade(let)s, notched tools and various other tools. The microburin technique was occasionally used for creating the distal truncation. However, the microburin index is low and the use of the microburin technique is not related to an increase of standardisation.

## Discussion

### E71K18C and other Afian assemblages

#### E71K18C and Makhadma 4

The site of Makhadma 4 in Middle-Egypt is attributed to the Afian complex [57]. Makhadma 4's occupation level is almost in direct contact with the Sheikh Houssein silts [58], previously interpreted as the results of catastrophic floods at the end of the Pleistocene (Wild Nile floods [59]) and now reinterpreted as lake shore deposits [20]. The site has yielded seven radiocarbon dates on charcoal ([35], see section above). Based on the hypothesis that E71K18 and Makhadma 4 are both related to the Afian industry, these dates have led Schild and Wendorf [21] to suggest a date of 16.5-15-14.5 cal ka BP for the Afian (and therefore E71K18).

When the characteristics of each lithic assemblage are compared, this attribution to the same complex remains to be demonstrated (see Table 10). Many differences are present between the two assemblages, as was previously noted by Vermeersch et al., p. 268 [57]: “Although there are many differences, the best typological fit for the Makhadma 4 assemblage is certainly the Afian, and it also fits within the same chronological period.” Both assemblages show that production was oriented towards obtaining short and wide elongated blanks which were then transformed into a variety of retouched tools, including proximal truncations and some geometrics. The Makhadma 4 assemblage is mostly composed of opposed platform cores, a relatively higher frequency of CTE's, including ridge blades and core tablets, striking platforms are mostly plain, there is no Levallois (or a few intrusive Levallois cores), and there are very few retouched tools, with an emphasis on burins. Some of these differences could be explained by a difference in the quality and availability of raw material, as previously suggested [57]. However, the characteristics of Makhadma 4 seem closer to those of E71K20 and therefore more similar to the Silsilian. There is not enough evidence to group Makhadma 4 and E71K18 under the same industry, thus the chronological attribution of the Afian based on the dates of the occupation at Makhadma 4 should be regarded with caution.

**Table 10:** Comparisons between E71K18C, E71K20, Makhadma 4 and GS-2B-II.

	E71K18C	Makhadma 4 –assemblage A [57]	E71K20	GS-2B-II [27]
**site settings**	Mostly surface site–no bone preserved, repetition of occupations ? (1) (2)	midden deposits–fish smoking and living area ?	Surface site	Stratified site—fill of minor Nile channel
**raw material**	wadi pebbles and small cobbles (1)	terrace cobles ; selection of pink and grey fine-grained chert + quartz	wadi pebbles and small cobbles (1)	chert and agate (4% debitage, 13% cores)
**general count**				
**cores**	334 (2%)	683 (3%)	148 (2%)	106 (2%)
**CTE's**	156 (1%)	232 (1%)	244 (3%)	34 (<1%)
**blades and fgts**	2240 (12%)	1835 (8%)	2037 (28%)	896 (18%)
**retouched tools**	1674 (9%)	171 (<1%)	423 (6%)	389 (8%)
**chips and flake fragments <2cm**	44%	NA (flakes + chips >80%)	15%	30%
**technique**	hard hammerstone (4)	hard hammerstone	hard / soft hammerstone	NA
**cores**				
**single platform**	11%	69%	25%	63%
**opposed platform**	40%	8%	46%	19%
**length**	mostly between 30 and 45mm long	mostly between 25 and 65mm long	mostly between 30 and 60mm long	mean length of around 25mm, range between 14 and 45mm
**characteristics of CTE's**	few, mainly overpassed products (44%)	mainly debordant (51%), ridge blades (35%) and core tablets (14%)	relatively numerous, mainly crested and overpassed products	NA
**elongated products**				
**mean length (sd)**	36.0mm (10.6)	43.1mm (18)	39.8mm (10.1)	NA
**mean width (sd)**	13.3mm (4.2)	16.1mm (7.7)	14.4mm (3.9)	NA
**mean thickness (sd)**	4.7mm (2)	6.7mm (6.1)	5.6mm (2.2)	NA
**mean length to width ratio (sd)**	2.8 (0.8)		2.8 (0.8)	45% between 2 and 3 and 40% between 3 and 4
**plain butts**	39%	70%	61%	71% (unfaceted)
**Faceted / dihedral butts**	39%	3%	7%	2%
**Linear / punctiform butts**	20%	24%	30%	
**mean butt thickness**	3mm (1.4)		2.4mm (1.5)	
**Unidirectional scar pattern**	63%		47%	
**Unidirectional and few opposed scar pattern**	10%		9%	
**Bidirectional scar pattern**	10%		18%	
**Retouched tools**	mainly microlithic, truncations, backed bladelets and geometrics	mainly burins (37%) and notched / denticulated tools (20%)	microlithic and macrolithic retouched tools, mainly backed pieces, especially distally backed	Mainly distal truncations (24%) and backed pieces (16%)
**backed retouch**	>65%	9%	>60%	16%
**removal of prox. Part for backed blade(let)s**	72%		50%	
**Imbt and Imbt restricted**	17 and 25	No microburin	5 and 8	12 and 37

(1) After Wendorf & Schild [24].

(2) After Close et al. [34].

#### E71K18C and site E-83-4 in Wadi Kubbaniya

Site E-83-4 is a surface scatter of artefacts, a rectangular area (5x6m) of which was selected for the collection of material [31]. All artefacts are eolised and the collected assemblage is small (461 pieces, including 262 chips and chunks and only 3 cores). The general characteristics of this (admittedly limited) assemblage resemble those of the Afian [31]. The artefacts were exposed on a deflated area and therefore bring no new information on the stratigraphic position of the Afian in the region.

### E71K20 and the other Silsilian assemblages

#### E71K20 and GS-2B-II

E71K20 is a surface site without available dates. However, based on the general characteristics of its assemblage, it was linked to site GS-2B-II in the Kom-Ombo Plain [27], attributed to the Silsilian and dated to around 14–15 kyr uncal BP. Table 10 compares the published data from GS-2B-II with the data from this analysis (E71K20). Their characteristics are very similar and justify their grouping, although (1) there is no evidence for the use of “exotic” material in E71K20, (2) single platform cores have a higher frequency at GS-2B-II, (3) blanks show a higher length to width ratio at GS-2B-II and (4) a lower frequency of distal truncations at GS-2B-II.

GS-2B-II is included within the Darau Member of the Gebel Silsila Formation [27]. Phillips and Butzer argue that a correlation with the Ballana–early Sahaba Formation of de Heinzelin [41] in Nubia is possible but cannot be demonstrated in the absence of data on textural, heavy mineral or clay analyses. Furthermore, according to Paulissen and Vermeersch [60] the Darau Member cannot be correlated with the Late Palaeolithic Alluviation of Wadi Kubbaniya. Dates from GS-2B-II are either on charcoal or shell but the date on charcoal was rejected, leaving one radiocarbon shell date of 12,440+/- 200 BC [27]. Radiocarbon dating on river mollusk shells are subject to errors ([61], p. 74). The chronological frame of the Silsilian industry therefore remains hypothetical.

#### E71K20 and Shuwikhat 2

Shuwikhat 2 is a small eroded surface site in the Makhadma area and is described by Vermeersch and colleagues [62]. More than 11000 artefacts have been collected. They are weathered with a light to intense patina. Core reduction is oriented towards the production of small (mean of 40mm long) and wide elongated blanks, mainly from opposed platform cores (80% of 107 cores). Ridge blades are present. Striking platforms of cores are plain (53%) or faceted (43%). Blanks show mainly punctiform (68%) or plain (23%) butts, associated with diffuse bulbs which would suggest the use of a soft hammer [62]. Retouched tools are not numerous and include endscrapers, Ouchtata bladelets, partially backed bladelets, notches and denticulates. Only a small number of truncations (N = 4) and basal blunting (N = 6) were noted. Whilst the high frequency of opposed platform cores distinguishes it from other Silsilian assemblages, it shows sufficient similar characteristics to justify its attribution to the Silsilian. However, its stratigraphic context could not be determined [62] and it therefore cannot contribute further to understanding the chronostratigraphical context of the Silsilian.

#### E71K20 and the Ballanan-Silsilian sites in Wadi Kubbaniya (E-78-5e and E-84-2)

E-78-5e was a surface concentration on the eroded surface of the Kubbaniyan silt [63]. Only part of the site was collected, comprising 2063 artefacts (including 1267 chips and chunks). It represents a blade industry with a high frequency of core trimming elements, and the use of various raw materials, dominated by chert and with a low frequency of agate, quartz, chalcedony, granite and jasper [63]. Cores are dominated by opposed platform cores; the most common types of butt among the debitage are plain and pointed. Retouched tools show a relatively high frequency of (mainly distal) truncations and scaled pieces. The use of the microburin technique is shown in the microburin traces on backed bladelets and truncations. Thus, despite the presence of scaled pieces, the characteristics of E-78-5e are comparable to those of E71K20.

E-84-2 is composed of several concentrations of artefacts contained in an indurated sand layer, just beneath the modern sand sheet. The artefacts are fresh or slightly eolised and likely to be in secondary position, reworked in a pond sediment by gravity and slopewash [64]. More than 9000 artefacts were recovered, both from the surface collection and from the excavation. They show a high frequency of blades, although these were not distinguished from flakes in the analysis of debitage [64]. The dominant butt type was plain for all debitage (around 70%). Core trimming elements are frequent. Single platform cores dominate (46%), followed by two-opposed platform cores (31%). Retouched tools include high frequencies of backed bladelets (>50%), truncations (mainly distal truncations) and notched tools (each 9%), and the occasional use of the microburin technique (mostly distal microburins). Blades with proximal retouch also occur. Overall this assemblage is therefore very close to the E71K20 assemblage. However, the geomorphological setting of the site is problematic [64] and, again, it does not add any significant information to the chronostratigraphical context of the Silsilian.

The Silsilian is sometimes grouped with the Ballanan, which was defined from three localities (Loc. 8956, 8957, 8863), near Ballana, in Egyptian Nubia [40]. This industry is defined as “microlithic and primarily made on blades, but there is no Levallois technology, the Halfa flake is missing, and there is a high frequency of bipolar technique in the production of blades and flakes. Furthermore, the major typological emphasis is on distal truncated microblades (…).”([40], p. 831). It is distinguished from the Silsilian by the frequent presence of the bipolar technique (cores on an anvil but with formed striking platforms). There also seems to be a high frequency of faceted striking platforms among the cores, dominated by single platform cores (nearly 50%), although butts on the blades were rarely identifiable; Wendorf suggests the use of the punch technique [40]. Retouched tools show high frequencies of truncations (mostly distal truncations), backed microblades and burins. Despite these differences, it seems likely that the Ballanan and the Silsilian are part of the same industrial complex, extending from Nubia into northern Upper Egypt.

### Variability in the Late Palaeolithic of the Nile Valley

Both assemblages are oriented towards the production of short and wide elongated unstandardised blanks, with a continuum between large and small blanks, manufactured from single and opposed platform cores showing a mainly unidirectional direction of core reduction (see Table 10). Both assemblages have yielded a high percentage of truncations and backed pieces and show evidence for an occasional use of the microburin technique. However, several characteristics distinguish them from each other: at E71K18C, cores are mainly exploited on their wide surface and the striking platforms are carefully prepared. Retouched tools include microlithic geometric tools and truncations are mainly proximal. At E71K20, single platform cores displaying a volumetric conception of debitage are frequent, striking platforms are simply prepared (plain butts) and maintained by the removal of core tablets. The convexities of the knapping surface are maintained through the removal of numerous core trimming elements, including ridge blades. Retouched tools include backed pieces, dominated by distally truncated blade(let)s but lack the microlithic geometric component of E71K18C.

The review of the geomorphological context of E71K18C, in tandem with the technological differences between Makhadma 4 and E71K18C, cast doubt on the chronology of E71K18 and the related Afian industry. If we assume a Late Pleistocene date for the Afian, this study highlights the technological variability of this period in the Nile Valley. Further analyses of other industries from the Late Palaeolithic in the Nile Valley (such as the Fakhurian, the Kubbaniyan, the Isnan or the Sebilian) will allow systematic comparisons between assemblages as well as a better consideration of this variability in the Nile Valley during the Late Palaeolithic.

### The Nile Valley at the end of the Pleistocene (between 25-12kaBP): Isolation of human populations?

One of the key questions for the Nile Valley at the end of the Pleistocene relates to the apparent isolation of populations from adjacent areas.

The characteristics from the two assemblages presented in this paper differ markedly from what is known in the Sudanese Nile Valley, namely at the site of Affad 23 where assemblages have been dated to 15–16 ka by Optically Stimulated Luminescence [65]. The numerous refittings at the site show the use of preferential Levallois methods for the production of large flakes, while retouched tools consist mainly of denticulates and scrapers. The authors attribute this assemblage to the Middle Stone Age (MSA), which makes it one of the latest occurrences of the MSA in Africa [65]. Although more data are needed, in the current state of knowledge, no link between the Middle Nile Valley technical behaviours and the Lower Nile Valley technical behaviours can be demonstrated for the end of the Pleistocene.

While affiliation with the eastern African record has been argued for earlier periods (e.g.[10]), the available record for the time period discussed here in eastern Africa is very sparse and many sequences document an occupational gap corresponding to MIS 2 (e.g. [66–71]). Further research on the cultural material from sites both in eastern Africa and in the Nile Valley is needed to improve our understanding of how human populations did (or did not) interact with each other at the end of the Pleistocene in this region.

Similarities with the Epipalaeolithic from the Levant, and particularly the Negev, have been suggested for assemblages from northern Sinai [72] and some recently published assemblages from Saudi Arabia (Nefud Desert, [73]). Early and Middle Epipalaeolithic assemblages of the Negev (~25–15 ka cal BP) are oriented towards the production of microliths, and can be generally characterised by the production of blade(let)s from single platform cores, with usually minimal core maintenance. Lithic variability is shown in different types of microliths, absence or systematic use of the microburin technique and technological variations in core preparation and maintenance [74–78]. While microlith types do not show obvious similarities, the presence of the microburin technique appears to be the most important shared feature between some Negev Epipalaeolithic assemblages and the Afian and the Silsilian. The use of the MBT is present at both Afian and Silsilian sites but these show a low microburin index (restricted IMbt below 26). The southern Levantine assemblages containing evidence for the use of the MBT usually show higher values of restricted IMbt (e.g. in the Nizzanan, Mushabian and Ramonian [75]). Most of the toolkits from the Late Palaeolithic assemblages from the Nile Valley include notched tools in relatively high frequencies. It thus remains possible that the use of the MBT in the Nile Valley is an independent innovation which could have come from “accidents” during the manufacture of notched tools. The assemblages studied in this paper therefore seem to belong to a different technical tradition than the Negev Epipalaeolithic. However, the analysis conducted in this study needs to be complemented in the future by the analysis of other Late Palaeolithic assemblages from the Nile Valley and by systematic comparative analyses of the lithic assemblages from the Nile Valley Late Palaeolithic and those from the Negev Epipalaeolithic, taking into account detailed technological characteristics. This may clarify whether other technological characteristics are similar between the two regions [79].

This paper analyses two lithic assemblages attributed to the Afian and to the Silsilian which allow discussion of Late Palaeolithic typo-technological variability in the Egyptian Nile Valley. This research complements previous studies that have focused mainly on stylistic and typological variability. It provides data that can be used for future comparative analyses. The Nile Valley is often presented as a ‘corridor’ and is key in the study of human dispersals. Comparative lithic analyses may help to contribute to discussing the archaeological visibility of dispersal hypotheses.

## Supporting information

S1 Appendix List of attributes and indexes used in the analysis. (DOCX)

S1 Database Raw data for lithic artefacts from E71K18C and E71K20. (XLSX)

S1 Table E71K18C –Supplementary tables. (XLSX)

S2 Table E71K20 –Supplementary tables. (XLSX)
