# Supplementary material for: Technological variability in the Late Palaeolithic lithic industries of the Egyptian Nile Valley: The case of the Silsilian and Afian industries
Source: PLoS One. 2017 Dec 27;12(12):e0188824. doi: 10.1371/journal.pone.0188824 (PMC5744920; doi:10.1371/journal.pone.0188824)
Supplement: S1 Appendix — (DOCX) [file pone.0188824.s001.docx]

| List of the main variables used in the lithic analysis | | |  |
| --- | --- | --- | --- |
|  |  |  |  |
| **For all:** |  | **For all elongated and core trimming elements** | |
| Provenance |  | Platform type | *plain* |
| Artefact number |  |  | *faceted* |
| determination | *elongated* |  | *dihedral* |
|  | *flake* |  | *linear or punctiform* |
|  | *core trimming element* |  | *broken* |
|  | *core* |  | *indet* |
|  | *retouched* | Platform breadth |  |
|  | *chip (<2cm)* | Platform depth |  |
|  | *chunk* | Bulb type | *absent* |
| fragmentation | *complete* |  | *slightly prominent* |
|  | *prox* |  | *prominent* |
|  | *dist* |  | *very prominent* |
|  | *mes* |  | *broken* |
|  | *lat* | Number of removal scars | |
|  | *multiple* | Scar pattern | *unidirectional* |
| Fragmentation | *complete* |  | *unidirectional and lateral* |
| Estimate of segment length  raw material | *Fragment* |  | *Bidirectional* |
|  | *Complete* |  | *Unidirentional and few opposed* |
|  |  |  | *centripetal* |
|  | *Small fragment* |  | *Other / indet* |
|  | *Almost complete* | Scar pattern morphology (orientation of the pieces with the platform at the bottom), see fig below | *unique array* |
| fire traces | *Yes / no* |  | *Y* |
| cortex | *A – none* |  | *parallel* |
|  | *B - < 33%* |  | *inverted Y* |
|  | *C – 33-66%* |  | *“fish bones”* |
|  | *D – 66-99%* |  | *“checked pattern”* |
|  | *E – 100%* |  | *“radiating”* |
| max length (technological) | | Longitudinal profile | *flat* |
| max width (perpendicular to length) | |  | *slightly curved* |
| max thickness | |  | *curved* |
| Technological determination / remark | |  | *very curved* |
|  | |  | *irregular* |
|  |  | Lateral profile | *flat* |
|  |  |  | *twisted* |
|  |  |  |  |
|  |  |  |  |
|  |  | edge morphology (only for (almost) complete blanks) – synthesis of data noted for each proximal, mesial and distal segments of blank | *Mostly rounded* |
|  |  |  | *Mostly parallel* |
|  |  |  | *distally convergent* |
|  |  |  | *convergent* |
|  |  |  | *Mostly divergent* |
|  |  |  | *other* |

| **For all cores** |  | **For all retouched** |  |
| --- | --- | --- | --- |
| General determination | | Typology |  |
| number of striking platforms | | Type of blank |  |
| number of debitage surfaces | | Platform type | *plain* |
| type of blank |  |  | *faceted* |
| type of striking platform | *plain* |  | *dihedral* |
|  | *faceted* |  | *linear or punctiform* |
|  | *dihedral* |  | *broken* |
|  | *cortical* |  | *indet* |
| type of preparation of debitage surface | *unipolar* |  | *removed* |
|  | *bidirectional* | Bulb thinning | Yes / no |
|  | *centripetal* | number of retouch |  |
|  | *indet* | location of retouch | (on which edge(s)) |
| type of debitage | *unidirectional* | type of retouch | *obverse* |
|  | *bidirectional* |  | *inverse* |
|  | *unidirectional and lateral* |  | *alternate* |
|  | *bidirectional* |  | *alternating* |
|  | *centripetal* |  | *bifacial* |
|  | *lineal (in case of Levallois)* |  |  |
| type of products | *flakes, elongated* |  |  |
| length of last removal |  |  |  |
| width of last removal |  |  |  |
| Type of core (number of striking platforms and debitage surfaces) |  |  |  |
|  |  |  |  |
|  |  |  |  |
|  |  |  |  |
|  |  |  |  |

**Ratios**
**Elongation index (ratio length/width)**
elongated blank Ratio ≥2

Flake Ratio <2

**Microburin indexes [1]**

Microburin index (Imb) $\left( \frac{\left( nb microburins \right)}{\left( nb microburins+nb retouched tools \right)} \right)* 100$

Restricted microburin index (Imbr ) $\left( \frac{nb microburins}{nb microburins+nb backed tools} \right)* 100$

**Figure 1**. Schematic representations of the different types of morphology of arrays or types of scar patterns.


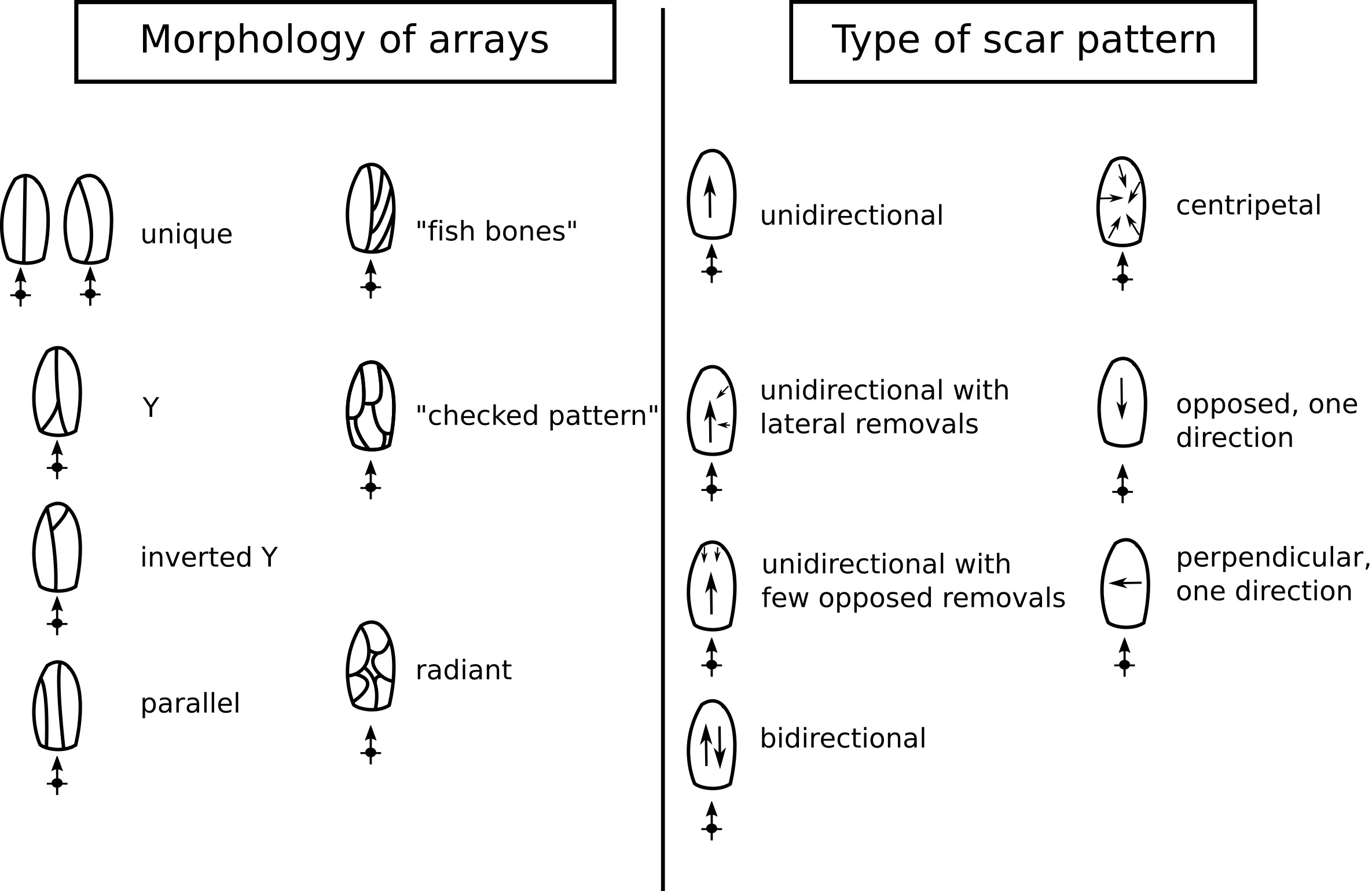


**References**

1. Henry DO. The utilization of the microburin technique in the Levant. Paléorient. 1974;2: 389–398.
